# Engaging community pharmacy in tuberculosis case detection (ENHANCE): a study protocol for an implementation study in Indonesia

**DOI:** 10.1186/s13690-025-01610-7

**Published:** 2025-05-07

**Authors:** Ivan Surya Pradipta, Carla Florencia, Cut Ainul Mardhiyyah, Firda Shafira Nurfadila, Kevin Aprilio, Rizky Abdulah, Bony Wiem Lestari, Rovina Ruslami, Trisasi Lestari, Rob Aarnoutse

**Affiliations:** 1 Rational Use of Medicine in Tuberculosis (RUNNERS) Research Group, Department of Pharmacology and Clinical Pharmacy, Faculty of Pharmacy, Universitas Padjadjaran, Bandung, Indonesia; 2 Drug Utilization and Pharmacoepidemiology Research Group, Center of Excellence in Higher Education for Pharmaceutical Care Innovation, Universitas Padjadjaran, Sumedang, Indonesia; 3 Doctoral Program of Pharmaceutical Science, Faculty of Pharmacy, Universitas Padjadjaran, Bandung, Indonesia; 4 Faculty of Pharmacy, Universitas Yayasan Pendidikan Imam Bonjol Majalengka, Cirebon, Indonesia; 5 Master Program of Pharmacoepidemiology and Pharmacoeconomics, Faculty of Pharmacy, Universitas Padjadjaran, Bandung, Indonesia; 6 Department of Public Health, Faculty of Medicine, Universitas Padjadjaran, Bandung, Indonesia; 7 Department of Biomedical Sciences, Division of Pharmacology, Faculty of Medicine, Universitas Padjadjaran, Bandung, Indonesia; 8 Center for Tropical Medicine, Faculty of Medicine, Public Health, and Nursing, Universitas Gadjah Mada, Yogyakarta, Indonesia; 9 Department of Pharmacy, Radboud Institute for Medical Innovation, Radboud University Medical Center, Nijmegen, The Netherlands

**Keywords:** Pharmacy, Tuberculosis, Implementation science, Public-private mix

## Abstract

**Introduction:**

A significant number of possible tuberculosis (TB) missing cases are still reported globally. Pharmacies are reported as a significant first point of contact for people with TB. Unfortunately, the practice of TB detection in pharmacies is still lacking. Therefore, this study aims to implement and evaluate a community pharmacy program for TB case finding in a systematic and structural approach.

**Methods:**

An implementation study will be piloted in Bandung City, Indonesia, from February to November 2025. The program will engage pharmacy personnel in screening, educating, and referring people with presumed TB to community health centers (CHCs) for further diagnostic work-up. This study will involve selecting 20 pharmacies and 4 CHCs. Sequential research activities will be performed, incorporating quantitative and qualitative approaches, i.e., (1) building a coalition, (2) developing a conceptual program, (3) program socialization and educational intervention, and (4) program implementation and evaluation. The program outcomes will be reached according to the sequential research activities: (1) a joint agreement among the key actors and implementers, (2) a conceptual program for implementation, (3) improved capacity of implementers and availability of practice aids and system for the implementation, (4) the effectiveness of the program implementation. The Consolidated Framework for Implementation Research will be used as a framework in this study. Descriptive and multivariable analyses will be used for quantitative data, while thematic analysis will be used for qualitative data. Finally, an implementation outcome will be comprehensively analyzed, considering the quantitative and qualitative data analyses for the key factors of the successful program.

**Supplementary Information:**

The online version contains supplementary material available at 10.1186/s13690-025-01610-7.

## Significance

This study will generate new knowledge about the impact of community pharmacy practice in increasing TB detection in a high TB-burden setting. This study will also provide a systematic and structural approach that can be replicated in other areas with similar characteristics.

**Supplementary Information:**

The online version contains supplementary material available at 10.1186/s13690-025-01610-7.

**Table:** 

**Text box 1. Contributions to the literature**	
• Increasing tuberculosis (TB) case finding has been a major concern in high-prevalence TB countries.	
• Pharmacies are reported as a significant first point of contact for people with TB but lack engagement for TB case findings.	
• This study highlights a systematic approach to engage pharmacies as a potential resource in increasing TB case findings.	
• Structural and collaborative approaches are described in this study protocol to develop a successful implementation program in increasing TB case findings.	
• This study will highlight the role of community pharmacy practice in increasing TB case finding, especially in a high-TB burden country.	

## Background

Global data showed that TB notifications dropped significantly [1], and other studies predicted an increase in TB incidence and mortality in the post-pandemic period [2]. Globally, Indonesia has the second rank of countries with the highest TB prevalence at 1.1 million estimated TB cases in 2023 [1]. Only 821.2 thousand of those cases were successfully notified, which indicates many TB missing cases in Indonesia [1]. West Java is one of the Indonesian provinces that has the highest TB prevalence in Indonesia, with around 211 thousand cases notified [3]. Comprehensive efforts are needed to increase TB case findings to accelerate national and global TB targets [4, 5].

A previous study in West Java, Indonesia, demonstrated that 40 per cent of people with TB come to pharmacies as the first point of contact for their TB symptoms (e.g., coughing, fever, and pain) prior to their diagnosis [6]. However, this group of people experienced an average delay in treatment initiation of 12–47 days [6]. These findings were also supported by a nationwide study highlighting the idea that community pharmacies can play a significant role in TB case detection through improved engagement in the national TB program [7].

Engaging all potential resources for TB control is strongly recommended by the World Health Organization (WHO). The involvement of pharmacy personnel in managing TB cases has been endorsed by the consensus between the WHO and the International Federation of Pharmacy (FIP) in 2011 [8], encouraging pharmacy personnel to actively find TB cases in the community. Our recent systematic scoping review highlighted that pharmacy personnel can indeed act as TB patient detectors in their community pharmacies by screening and referring visitors to a health facility for further TB examination [9]. Previous studies from Pakistan [10], Vietnam [11], and Bolivia [12] have revealed that community pharmacy personnel can support TB case findings in the community, especially in high-burden TB countries.

Unfortunately, the practice of pharmacy personnel in TB detection is still lacking in Indonesia. A previous nationwide survey among 1,129 pharmacy personnel showed that only 2 per cent of pharmacy personnel regularly screened for people with presumed TB [13]. Lack of TB educational exposure was identified as the main factor that led to suboptimal TB knowledge and awareness of TB practices among community pharmacy personnel [13, 14]. The study also identified that half of the participants had never received TB training, while the majority of participants who had been exposed to TB training received their TB training more than two years ago. Moreover, our qualitative study explored the fact that pharmacy personnel were not aware of the national TB epidemic, since no systematic and structural involvement for pharmacy personnel was provided in the national TB program [15, 16]. The lack of clear direction and guidance for pharmacy personnel led to their disengagement in TB case finding [15, 16].

Although earlier studies conducted outside of Indonesia focused on the relevance of pharmacy TB case detection [17–19], their study did not explicitly outline systematic and structural approaches to involving pharmacies in the implementation. It is further important to take account of the local situation when engaging with pharmacies. Therefore, we propose an implementation study for community pharmacies on TB detection in a high-burden TB country.

The overall aim of this study is to develop, implement, and evaluate a program involving community pharmacies in TB detection in Indonesia with several specific objectives: (1) to build and encourage a strong coalition among relevant resources for the program implementation; (2) to develop a conceptual pharmacy model for TB case detection based on the local context; (3) to socialize the program with the key actors, implementers, and stakeholders, as well as to improve the capacity of pharmacy personnel in TB detection; and (4) to implement and evaluate a pilot of the program. This study will provide a unique approach that not only improves the capacity of the pharmacy personnel in TB but also builds networks and systems among pharmacies, local TB programmers and professional organizations for contributing to effective and sustainable active TB case-finding practice in the community.

## Methods and design

### Context setting

The study will be conducted in Bandung, West Java’s capital city with a total population of 2.5 million people [17]. The study will be performed from February to November 2025. Bandung is one of the areas with the highest TB prevalence in West Java [18], and community health centers (CHCs) are the backbone facilities for managing TB cases. CHCs were established as the primary public health facility at the sub-district level under the authority of the local government [19]. CHCs are commonly surrounded by community pharmacies.

In Indonesia, pharmacies are part of the private sector operated by at least two pharmacy personnel, i.e., a responsible pharmacist and a pharmacy assistant [14]. The responsible pharmacist must hold at least a professional degree in pharmacy, while the pharmacy assistant must have completed at least a pharmaceutical vocational school. Both of them must hold a practicing license from the government [14]. According to the municipal regulations, a CHC serves as a supervisor for the surrounding pharmacies, and pharmacies are responsible for supporting CHC activities aimed at promoting community health [19]. This regulatory framework allows pharmacy personnel at the CHC to build networks the CHC with surrounding pharmacies for the TB case detection program.

In Bandung, 11 of 80 CHCs have a rapid molecular test instrument for TB diagnostic tests, with around 900 pharmacies being registered with the local government. We will use purposive sampling in this study, considering the potential funding and resources available. Four CHCs will be selected according to the highest TB prevalence and availability of TB services and resources (i.e., medical doctors, nurses, TB analysts, and a TB laboratory equipped with rapid molecular tests). Five pharmacies per CHC will be the target site for implementing the TB case detection program selected based on the following criteria: (1) committed pharmacy personnel to follow all phases of the program proved by a commitment letter; (2) located near the targeted CHC; (3) providing service for prescription and non-prescription medicine; and (4) having a minimum of 60 non-prescription visitors per day. While no regulations enforce the participation of each pharmacy in this program, we offered several incentives as a form of encouragement, such as free TB training with a formal certificate, transportation fees, and credit points for continuing their practice license. Considering the population density and daily pharmacy visits that can reach 30 visitors per day, around 54,000 visitors will be the potential screening target for 3 months of program implementation.

### Study design

Subsequent activities will be performed with several approaches and study designs. An active TB case-finding program will be developed and implemented in the pilot setting using qualitative, quantitative and interventional approaches, using the Consolidated Framework for Implementation Research (CFIR) as a study framework [20]. The CFIR framework will focus on essential domains for an implementation study, such as intervention characteristics, outer settings, inner settings, characteristics of individuals, and implementation processes. Assessing the domains will be used to mitigate potential problems, understand the implementation process and evaluate the program implementation [20].

### Program implementation

**Fig. 1:**
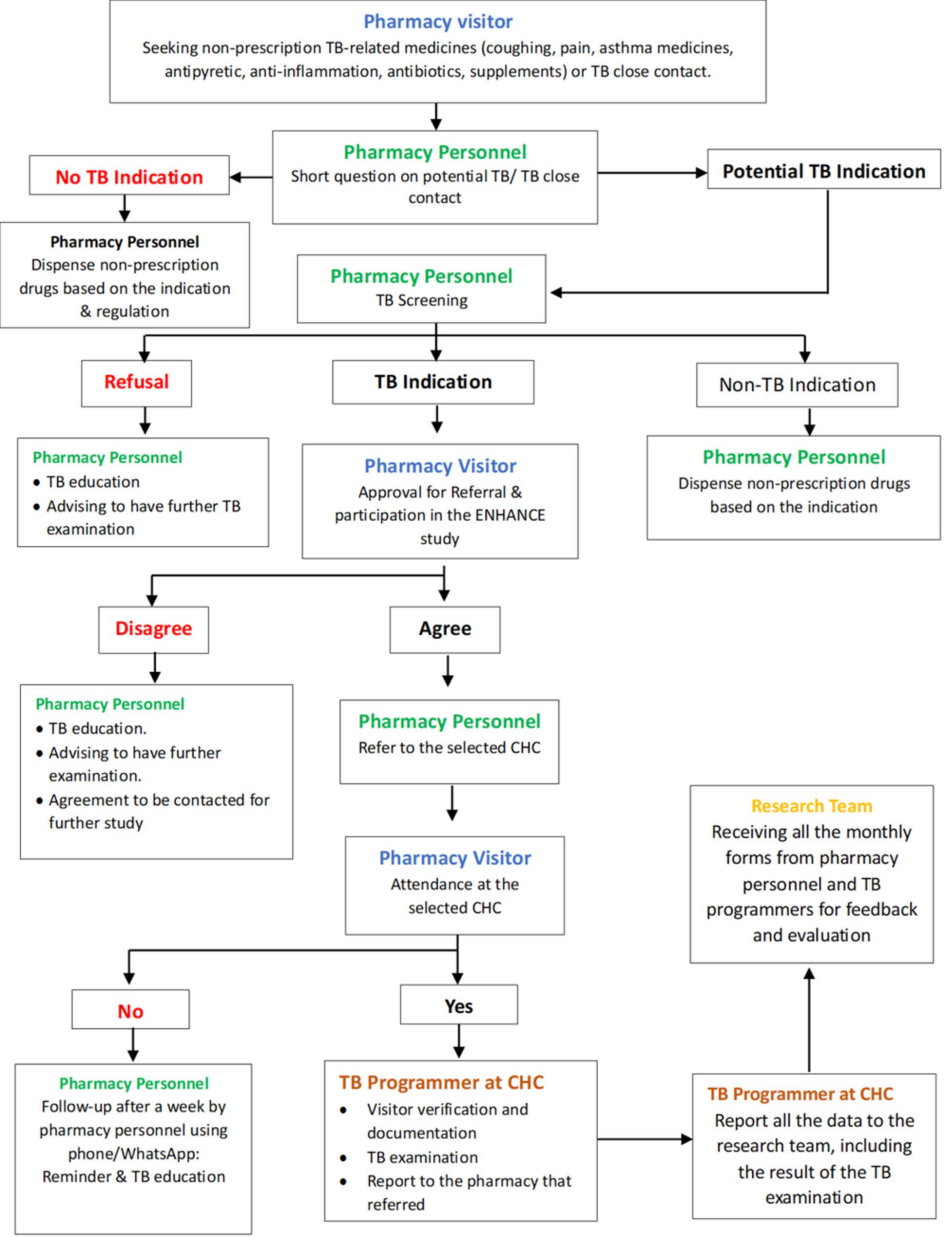
Pharmacy visitor pathway within the framework of the engaging community pharmacy in tuberculosis case detection (ENHANCE) study, conducted in Bandung, Indonesia from February- November 2025

The program will focus on the activities of pharmacy personnel at selected pharmacies in TB case detection. The pharmacy personnel should screen existing TB symptoms of visitors by asking about their symptoms (e.g., coughing for more than 2 weeks, sweating during the night, decreasing body weight, existing TB close contacts), and then referring them to a selected CHC for further TB examination. Not all visitors will be screened; only visitors with indications of possible TB will be screened, i.e., visitors who buy coughing drugs, analgesics, antibiotics, asthma medicines, and dietary supplements, and visitors known to have contacted people with TB from medication records or direct communication. Once the visitor is indicated as a person with presumed TB, pharmacy personnel will refer the visitor to the selected CHC for further TB examination using a pharmacy referral letter. After the referral letter is given to the visitor, the pharmacy personnel will ensure the visitor goes for a further TB examination at the referred CHC through phone contact or short messages. Visitors who refuse the screening at the pharmacy and who are lost to follow-up from the pharmacy to CHC will be reminded and provided educational exposure about the importance of TB screening through phone contact or an attractive online leaflet. The pharmacy visitor’s pathway is described in Fig. 1.

This study will include essential stakeholders, key actors, and implementers. Stakeholders are institutions and organizations involved in program implementation, such as the local health district office, which manages community health centers, and professional organizations (i.e., pharmacist and pharmacy assistant organizations), which organize community pharmacy personnel. Key actors are those with the capacity to mobilize implementers and relevant stakeholders for the implementation study, such as the coordinator of local TB programmers and pharmaceutical services at the local district health office; representatives of TB programmers and pharmacists at the CHC level; and representatives of local professional organizations. The implementers are people who will directly implement the program; they include a TB programmer, a pharmacist from the selected CHCs, and pharmacy personnel from the selected pharmacies.

### Procedures and outcomes

In line with the study objectives, the study will consist of four phases with several outcomes:

#### Phase I: Building a strong coalition

The objective of this phase is to engage all potential resources and establish a strong coalition or task force consisting of key actors with a clear role and responsibility for program implementation. We will conduct focus group discussions (FGDs), in-depth interviews (IDIs) and informal discussions with the key actors to explore the potential implementation of the program and their roles in its implementation. The outcome of this phase will be a joint statement and agreement for program implementation among the key actors and implementers. This phase will be conducted from February to March 2025.

#### Phase II: Development of the conceptual model

The objectives of this phase are: (1) to adapt the theoretical model of the program into the local context, (2) to mitigate potential barriers in the program implementation; and (3) to develop intervention packages for successful program implementation. A qualitative study with a case study design [21] will be performed by involving key actors, implementers and experts in the areas of community pharmacy, public health, and implementation science to criticize the theoretical model for pharmacy TB case detection previously developed by literature studies. The adaptation process will include several aspects, i.e., the screening item and process, visitor pathways, communication system, educational material, reminder system, participant roles, documentation, reporting and technical issues. The FGD will be performed to adapt the theoretical model and mitigate potential barriers to the program implementation [20]. The FGD will consist of 7 participants with diverse backgrounds, such as pharmacists, pharmacy assistants, TB programmers, professional organizations and pharmacists at the CHC level, a TB coordinator at the district level and representatives of the public. If there is still information needed, we will conduct another FGD or individual interviews until all information is comprehensively received. The interviews and discussions will start with broad questions, i.e., “why do people with TB get delayed TB treatment?”, “what is, in your opinion, the optimal role of pharmacy personnel in TB case detection?”, “what do you think about the responsibility of pharmacy personnel for TB case detection?”, “why are pharmacy personnel not optimal in TB case detection?”, “what should be prepared to have a TB case detection program for pharmacy personnel?”, and “how is the effective flow for TB case detection that involves pharmacy personnel?”. After the participants discussed these questions, we will propose a theoretical model to receive feedback from the participants. The study outcome of this phase will be an adaptable pharmacy program on TB detection based on the local context and a package of interventions to support the successful implementation of the program. This phase will be conducted from April to May 2025.

#### Phase III: Program socialization and capacity Building

The objective of this phase is to: (1) socialize the program concept and its implementation to the key actors and implementers, (2) improve the capacity of the implementers for the program implementation, and (3) receive a strong commitment from the implementers for the study’s implementation. We will provide a two-day certified workshop to the key actors and implementers. The workshop will cover topics related to the program concept and system, communication skills for TB screening, and TB training for pharmacy personnel on TB case detection. We will perform a pre-and post-test evaluation to analyze the improvement of participants’ knowledge and their perceptions related to the TB case detection program. At the end of the workshop, a joint commitment for the program implementation will be signed among the implementers related to the role, rights and responsibility in the study implementation. The study outcome of this phase will be the improvement of knowledge, perception and skills of the implementers related to the TB case detection and implemented program and a joint commitment statement from the implementers. This phase will be conducted from June to August 2025.

#### Phase IV: Program implementation and evaluation

The objective is to implement and evaluate the developed program based on the local context. Considering available resources and a feasibility study for a newly developed program, we will conduct an implementation study at the pilot site for 3 months. The implementers will perform activities related to TB case detection using the adapted model produced from phase II. To mitigate potential dropouts or inactive pharmacies, we will perform biweekly onsite monitoring to provide technical supervision and ensure the pharmacies can perform the program. We will also provide a monthly meeting between the research team and implementers to collect data needed for the study, discuss the retention and application of knowledge that was provided in the training, and seek barriers and facilitators in the program’s practice.

A mixed methods approach will be used to evaluate the program implementation. We will perform a quantitative analysis to analyze the effectiveness of the program based on the monthly visitor database in the CHC from the referral process (i.e., the identity of visitors referred by the pharmacy and the referring pharmacy; date of the referral letter, visit to the CHC, and TB examination in the CHC; type of TB examination and final medical decision at the CHC). The responsible person for the CHC will then report the activity to the research team using the monthly report form in the monthly meeting.

As part of the monthly meeting, we will use a qualitative approach to explore the process, barriers, and facilitators of the program implementation. We will perform FGDs with program implementers in the first, second, and third months period of the program implementation. The FGD will be conducted for selected implementers who successfully and unsuccessfully follow all steps of the program. We will perform 4 rounds of FGD; each FGD will consist of a maximum of 5 participants. To explore visitor perspectives, in-depth interviews (IDIs) will be performed with a total of 5–10 visitors who are lost to follow-up and non-lost-to-follow-up to explore their convenience, satisfaction, barriers and facilitators regarding TB screening, and access to health facilities for further examination and diagnosis process. All feedback will be given by the research team to implementers in the monthly meeting to improve TB case detection practice in the real world. All quantitative and qualitative data will be collected by the research team during program supervision in the first, second, and third months of the program implementation. The data from implementers will be summarized using a monitoring form. Phase IV will be conducted in September-November 2025. All the study phases are summarized in Table 1.

**Table 1:** Phases of the engaging community pharmacy in tuberculosis case detection (ENHANCE) study, conducted in Bandung, Indonesia from February- November 2025

Study Phase	Phase’s title	Objectives	Activities	Outputs	Timeline
I	Building coalition	To engage all stakeholders and key actors for successful program implementation	1. FGDs 2. IDIs 3. Informal discussion	A joint agreement and statement of key actors to participate in the program implementation	February-March 2025
II	Program development	1. To adapt the theoretical conceptual model of the program into the local context 2. To mitigate potential barriers in the program implementation; and 3. To develop intervention packages for the successful implementation of the program.	1. Literature Review 2. IDIs 3. FGDs	1. An adapted conceptual model for program implementation 2. Program guidance, training material and practice aids for the program implementation	April-May 2025
III	Socialization and capacity building	1. To socialize the program concept and its instrument aids to the key actors and implementers. 2. To improve the capacity of the implementers for program implementation. 3. To receive a strong commitment from the implementers for the study’s implementation.	A two-day certified workshop	1. Improving the capacity of implementers on knowledge and skills related to TB case detection 2. A joint agreement and statement of the implementer to participate in the program implementation	June-August 2025
IV	Implementation and Evaluation	1. To implement the developed program 2. To evaluate the developed program	1. A three-month program implementation 2. Program monitoring and supervision 3. FGD	1. Effectiveness of the program, i.e., (1) the number of pharmacy visitors who receive TB screening by pharmacy personnel; (2) the number of pharmacy visitors with presumed TB referred to the CHC; (3) the number of pharmacy visitors who receive TB examination; and (4) the number of pharmacy visitors who are TB positive 2. Implementation program, i.e., key factors of success in the program implementation	September-November 2025

Note: FGDs: Focus Group Discussions; IDIs: In-depth Interviews

### Data management and analysis

In qualitative studies from phases I, II, and IV, we will record and transcribe all interviews and FGDs for data analysis. All data will then be transferred to a qualitative data analysis software package, ATLAS.ti version 8 [22]. The data will be stored on a computer protected by a specific username and password. Only two coders for qualitative data analysis (data analysts) and the principal investigator (PI) can access the qualitative data. The qualitative data will be anonymously analyzed using thematic analysis following aspects of the CFIR framework [20]. The PI will resolve all discrepancies during the data analysis between the two coders. Triangulation procedures for qualitative and quantitative data will be performed to increase validity of the findings. The quantitative data will show the trend of TB case-finding activities among implementers, while the qualitative data will provide insights into why or how that pattern exists. Moreover, peer debriefing will also be conducted among the key actors, research team, and selected implementers to get feedback on the quality of the data and its analysis. We will discuss the conceptual model—including the screening algorithm, educational material, reminder, recording, and reporting system—with the FGD participants in Phase II. These findings will be used in each subsequent phase to validate the feasibility and effectiveness of the conceptual model for program implementation.

In Phase III, a pre-post quantitative data analysis will be conducted with the implementers from the educational program activity. The key success indicator for the educational intervention will be the improvement of the participants’ post-test scores. A previously validated instrument [23] will be used to assess the improvement of the participant’s knowledge and perception of TB detection. Since the conceptual model can potentially be revised based on activities in Phase II, we will develop the questionnaire related to the practical issues for the screening, referral, and communication process considering findings from Phase II. This questionnaire will then be validated with TB experts in the research team. A summative index will be assessed for knowledge, attitude and practical issues related to the TB case detection program. Participants should acquire an arbitrary minimum score of 85 out of 100 on the post-test to ensure acceptable competence in the areas of knowledge, attitude, and practice in TB case detection [23].

In Phase IV, participating pharmacies will record their referral data using a patient screening form (Additional file 1), and a copy of the referral letter to the CHC (Additional file 2) that will be kept in the pharmacy. The screening form and copy of the referral letter will be reported by pharmacy personnel to the researcher team using a monthly report form. Data for referral activities at the pharmacy will be entered by a data analyst in the research team based on the screening form and referral letter reported monthly by pharmacy personnel. The data analyst will perform data entry manually on a computer with a specific username and password. Only the data analyst and PI can access the laptop. All the manual datasets (i.e., report forms) will be descriptively analyzed from the first to the third month of implementation, including (1) the number of pharmacy visitors who received TB screening by pharmacy personnel; (2) the number of pharmacy visitors with presumed TB who were referred to the CHC; (3) the number of pharmacy visitors who received TB examination; and (4) the number of pharmacy visitors who were TB positive. A qualitative approach will be performed using the CFIR framework [20] that focuses on key factors of success in the program implementation, following the procedure and principles of the qualitative study described previously.

Considering ethical issues, all forms in the pharmacy and CHC will be safely stored in locked filing cabinets that can only be accessed by the implementers. These forms will be collected by the research team every week and subsequently be safely stored by the research team in a locked filing cabinet that can only be accessed by the PI. All softcopy data will be anonymously analyzed and stored on the PI’s personal computer protected by a password.

We will analyze all quantitative data using descriptive analysis. A paired sample *t*-test or the alternative non-parametric test will be used to analyze the effect of the educational program on knowledge and perceptions of TB detection activities and the program. A multivariable analysis will be used to analyze factors associated with capacity improvement among the participants in Phase II and with the practice of TB detection in Phase IV. Bivariate logistic regression will be used to determine the association between the independent and dependent variables. A *p*-value of < 0.25 will be applied as a threshold to include potential variables in the multivariable analysis. Logistic regression analysis will be used in multivariable analyses. All significance levels will be set at 5 per cent, and 95% Confidence Intervals (CIs) will be presented. The quantitative analyses will be conducted using SPSS version 26 [24]. Anonymized data will be applied in all the data management and analysis.

Finally, the effectiveness of TB case detection activity will be measured from the quantitative findings, i.e., (1) the number of pharmacy visitors who receive TB screening by pharmacy personnel; (2) the number of pharmacy visitors with presumed TB who are referred to the CHC; (3) the number of pharmacy visitors who receive TB examination; and (4) the number of pharmacy visitors who are TB positive. To understand and evaluate the comprehensive picture of program implementation, we will analyze qualitative findings to strengthen the program concept and identify any potential issues for scaling up the program to a broader population or location.

## Discussion

This study will use a simple and straightforward intervention, relatively cheap and feasible to be independently replicated in another area and local health offices. We indeed expect this program to be replicated based on the technical guidance developed and disseminated through this pilot-scale implementation.

This study does not consider a digital tool for screening, notification, and reporting processes since its use requires a high level of readiness. Our previous study identified that individual characteristics, ability to operate, cost, available facilities, and infrastructure all have varying effects on digital tool utilization [25]. Given our local context, we have decided to use a manual system for program implementers since it is less expensive and more easily implemented compared to its digital counterparts. We have further conducted an FGD to ensure the acceptability and convenience of our manual system for the program implementers. In practical aspects, we will additionally guidance throughout the utilization of the manual system.

Several challenges and limitations should be addressed for successful implementation. Firstly, pharmacy personnel are the key implementers of this project. Resistance from pharmacy professionals may occur as a result of adding new activities to their routine. It is vital to assess and intervene in pharmacy personnel aspects (knowledge, competence, and perception) during the capacity-building program (Phase III). To improve their willingness to participate in this initiative, we will provide education on their critical role in TB case finding and give supporting facilities, such as professional credit points to maintain their practicing license, transportation cost, free certified training, and advertisement of their pharmacies in social media as pioneers in the TB case-finding program. Secondly, refusal from pharmacy visitors to do further TB examinations. We will approach this issue by improving their tuberculosis awareness through effective communication and instruction materials provided by pharmacy personnel. The behavioral theory of health belief model [26] will be utilized in the educational material to improve the visitor’s perceived susceptibility, severity, benefit, and barriers. To prevent lost-to-follow-up visitors, the presumed TB visitor will be offered a free TB examination with no registration queue at the CHC. Thirdly, various other aspects should also be considered for successful implementation, including guidance, professional interaction, resources, and regulation [27]. We will address this issue in the implementation phase by providing practice guidance, developing a communication system among the implementers and establishing joint commitment from key actors and implementers. Fourthly, purposive sampling in this study may raise potential selection bias since this study did not consider the variability of pharmacy personnel and health infrastructure. However, since we intend this study as a feasibility study, we believe that all relevant factors can be further analyzed to strengthen further program implementation. Considering similar characteristics of the health system, structure, and regulation of pharmacies and CHCs in all areas of Indonesia, the generalizability of these findings will be high to other areas of Indonesia or even to other areas abroad that have similar characteristics.

As a unique approach compared to other similar studies [28–30], we will work together with key actors from the beginning of the study. This collaboration may facilitate the application and use of the program in the real world. The process of program supervision and monitoring will also be conducted together with the key actors and implementers. This approach will raise knowledge, awareness, and a strong coalition to implement sustainable practices in TB case detection.

## Electronic supplementary material

Below is the link to the electronic supplementary material.

Additional 1: file 1. Screening form. Additional 2: file 2. Referral Letter.

## Data Availability

No datasets were generated or analysed during the current study.

## Abbreviations

ENHANCE: Engaging Community Pharmacy in Tuberculosis Case Detection
TB: Tuberculosis
CHC: Community Health Center
CFIR: Consolidated Framework for Implementation Research
WHO: World Health Organization
FGD: Focus Group Discussion
IDI: In-Depth Interviews
PI: Principal investigator
CI: Confidence Interval
WHO-SEARO: World Health Organization-Southeast Asia Regional Office
