# Supplementary material for: Engaging community pharmacy in tuberculosis case detection (ENHANCE): a study protocol for an implementation study in Indonesia
Source: Arch Public Health. 2025 May 7;83:126. doi: 10.1186/s13690-025-01610-7 (PMC12057214; doi:10.1186/s13690-025-01610-7)
Supplement: Supplementary file 1 — Additional 1: file 1. Screening form. Additional 2: file 2. Referral Letter. [file 13690_2025_1610_MOESM1_ESM.pdf]

Additional file 1. Screening Form

SCREENING FORM
[Pharmacy Name]

Visitor's Identity

Name :
Date and Place of Birth :
Refusal from visitor to be screened : Yes/ No *)
If Yes, Please describe the reason : ……………………………………………………………..
……………………………………………………………..
……………………………………………………………..

No	Current Symptoms	Yes	No
1	Persistent cough with phlegm for more than 2 weeks		
2	Coughing up blood		
3	Fever/chills >1 month		
4	Night sweats		
5	Unexplained weight loss		
6	Lump in the neck area approximately 2 cm		
7	Shortness of breath and chest pain		

No	Condition	Yes	No
1	Contact with a tuberculosis patient		
2	Has diabetes mellitus		
3	Age >60 years		
4	Pregnant		
5	Smoker		
6.	History of tuberculosis		
7.	Currently taking other medications<br>If "Yes", specify the type of medication:<br>……………………………………..		

Who provided the information above?

☐ Direct medicine user
☐ Non-direct medicine users (e.g., friends, family or parents)

Should the visitor be referred for further examination?

☐ Yes
☐ No

Are the visitors willing to be referred to a community health centre (Puskesmas)?

☐ Yes
☐ No
☐ Other health facilities

Please specify:

- Visitor's address : …………………………………….
- Visitor's phone number : …………………………………….
- National Identification Number (NIK): ……………………………………
- The referred health facility :……………………………………..

Date of screening: ……………………..
Pharmacy staff

(……………………………..)

Additional file 2. Referral letter

REFERRAL LETTER

No. referral letter:

To :
Clinic/ hospital/ Community Health Center (Puskesmas) _________

Please conduct an examination for:

Name : ______________________
Age : ______________________
Gender : ______________________
Address : ______________________

Tuberculosis Screening Results by Pharmacy Personnel:

☐ Household contact ☐ Close Contact ☐ Other potential TB symptoms

Other Symptoms :

☐ Persistent cough/ coughing up blood
☐ Shortness of breath
☐ Night Sweats
☐ Fever/chills >1 month
☐ Unexplained weight loss
☐ Lump in the neck area approximately 2 cm

Risk Factors :

☐ Diabetes Mellitus
☐ Age >60 years
☐ Pregnant Woman
☐ Smoker
☐ Incomplete previous TB treatment
☐ Age <5 years

Thank you for your attention
__________,___/___/_____

Pharmacy personnel
